# Benzo[a]pyrene-derived quinones exploit mitochondrial electron transport to drive catalytic redox cycling and potent ROS amplification

**DOI:** 10.1007/s11010-026-05736-2

**Published:** 2026-09-15

**Authors:** Y. Robert Li, Hong Zhu, Michael A. Trush

**Affiliations:** 1 Duquesne University Nasuti College of Osteopathic Medicine, Pittsburgh, PA USA; 2 Johns Hopkins University Bloomberg School of Public Health, Baltimore, MD USA

**Keywords:** Benzo[a]pyrene-derived quinones, Complex I, Complex III, Hydrogen peroxide, Mitochondrial electron transport chain, Oxidative stress, Reactive oxygen species, Redox cycling, Superoxide

## Abstract

Benzo[a]pyrene (BP)-derived quinones are major oxidative metabolites of this ubiquitous environmental carcinogen, yet the mechanisms by which they generate reactive oxygen species (ROS) remain poorly understood. Here, we tested the hypothesis that BP-derived quinones exploit mitochondrial electron transport to undergo redox cycling and amplify ROS production. Using isolated mitochondria energized with either pyruvate/malate or succinate, we found that BP-1,6-quinone (BP-1,6-Q), BP-3,6-quinone (BP-3,6-Q), BP-6,12-quinone (BP-6,12-Q), and BP-7,8-quinone (BP-7,8-Q) stimulated potassium cyanide-resistant oxygen consumption, indicating active mitochondrial redox cycling. BP quinone redox cycling was accompanied by substantial production of superoxide and hydrogen peroxide, with BP-1,6-Q, BP-3,6-Q, and BP-6,12-Q exhibiting the greatest activity. Notably, BP-1,6-Q induced robust ROS generation at submicromolar concentrations, suggesting that mitochondria can serve as highly efficient amplifiers of BP quinone-mediated oxidative stress. Pharmacological dissection of the mitochondrial electron transport chain suggested complexes I and III as the principal sites supporting BP quinone redox cycling and ROS production, whereas the contributions of complexes II and IV were negligible. These findings indicate that BP-derived quinones exploit electron flow through mitochondrial complexes I and III to drive catalytic redox cycling and potent ROS amplification. This previously underappreciated mechanism provides a mechanistic link between BP metabolism, mitochondrial oxidative stress, and the pathogenesis of BP-associated toxicity.

## Introduction

Benzo[a]pyrene (BP) is a ubiquitous environmental pollutant generated by industrial activities, fossil fuel combustion, and tobacco smoke, and is also present in contaminated foods [1, 2]. Extensive studies have established BP as a potent carcinogen [3], and exposure to BP has also been associated with other toxicities including teratogenesis, hematotoxicity and adverse cardiovascular effects [4–7]. Although the mechanisms underlying these diverse toxicities remain incompletely understood, many appear to depend on metabolic activation of BP.

**Fig. 1:**
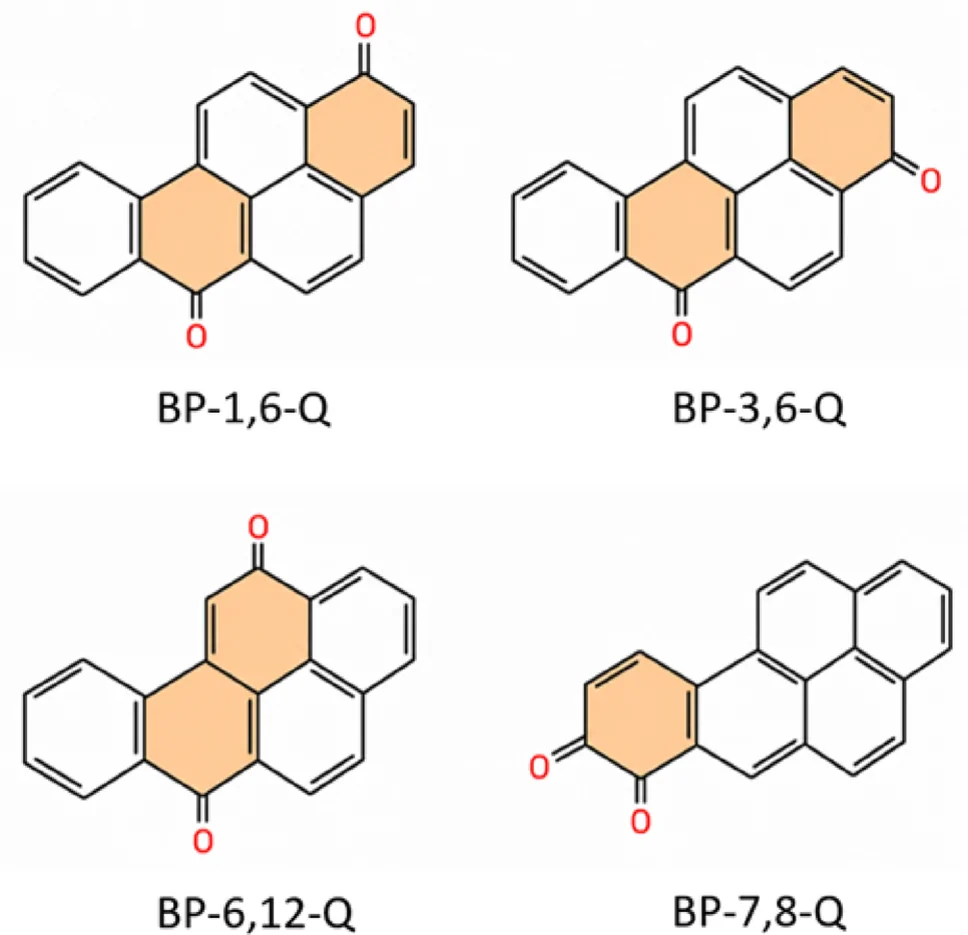
Structures of BP-derived quinones

BP undergoes extensive oxidation by cytochrome P450 enzymes to generate numerous metabolites [3]. While considerable attention has focused on the formation of the carcinogenic BP-7,8-diol-9,10-epoxide (BPDE), BP metabolism also produces substantial quantities of quinone metabolites, including BP-1,6-quinone (BP-1,6-Q), BP-3,6-quinone (BP-3,6-Q), BP-6,12-quinone (BP-6,12-Q), and BP-7,8-quinone (BP-7,8-Q) [3, 8, 9] (Fig. 1). Formation of these quinone metabolites has been demonstrated in numerous cell types and tissues, and they frequently account for up to 50% of total BP metabolites [10–12]. Thus, quinone formation represents a major metabolic fate of BP and may contribute importantly to its biological effects.

Quinones are well-recognized mediators of cellular injury that exert toxicity through electrophilic reactions with biomolecules and/or through redox cycling-mediated generation of reactive oxygen species (ROS) [13, 14]. Previous studies have shown that BP-derived quinones can form DNA adducts and induce cytotoxicity at low micromolar concentrations [15–18]. The O_2_ dependence of quinone-induced cytotoxicity further implicates ROS as an important mediator of their biological effects [16]. However, the exact cellular sources and detailed mechanisms responsible for ROS generation by various BP-derived quinones remain partially understood [19, 20].

Mitochondria are a major intracellular source of ROS and contain multiple redox-active enzymes capable of catalyzing one-electron reduction of quinones. Previous studies from our laboratory demonstrated that BP-derived quinones significantly alter mitochondrial morphology and function [18], suggesting that mitochondria may represent a critical intracellular site for quinone redox cycling. We hypothesized that BP-derived quinones exploit electron flow through the mitochondrial electron transport chain (METC) to undergo catalytic redox cycling, thereby amplifying ROS production. In the present study, using mitochondria isolated from human monocytes/macrophages, we investigated the ability of various BP-derived quinones to undergo redox cycling within the METC and characterized the resulting ROS generation. Our findings indicate that mitochondrial complexes I and III are major catalytic sites of BP quinone redox cycling and support substantial ROS amplification at submicromolar quinone concentrations. These results suggest a previously underappreciated mechanism by which environmental quinones hijack mitochondrial electron transport processes to promote oxidative stress and toxicity.

## Materials and methods

### Materials

BP-1,6-Q, BP-3,6-Q, BP-6,12-Q and BP-7,8-Q were obtained from the Midwest Research Institute (Kansas City, MO). Lucigenin, luminol, rotenone (ROT), antimycin A (AA), myxothiazol (MYX), diphenyleneiodonium (DPI), succinic acid (sodium salt), pyruvic acid (sodium salt), maleic acid (disodium salt), Cu, Zn superoxide dismutase (SOD) from bovine erythrocytes, catalase from bovine liver, horseradish peroxidase (HRP) (type II), and bovine serum albumin (BSA) were purchased form Sigma Chemical Co. (St. Louis, MO). Tetramethyl-1,4-phenylenediamine (TMPD), thenoyltrifluoroacetone (TTFA), and potassium cyanide (KCN) were products of Aldrich Chemical Co. (Milwaukee, WI). 5-(Dimethoxyphosphoryl)−5-methyl-1-pyrroline-*N*-oxide (DEPMPO) was purchased from Oxis International (Portland, OR). RPMI 1640, Dulbecco’s phosphate-buffered saline (PBS), penicillin and streptomycin were products of Life Technologies (Grand Island, NY). Fetal bovine serum (FBS) was obtained from Biowhittaker (Walkersville, MD). Tissue culture flasks were purchased from Corning Costar Co. (Cambridge, MA).

### Culture and differentiation of ML-1 cells to monocytes/macrophages

Human monoblastic ML-1 cells were obtained from Dr. Ruth W. Craig, Dartmouth Medical School, NH. The cells were cultured at 37 °C in an atmosphere of 5% CO_2_ in RPMI 1640 medium supplemented with penicillin (50 units/ml), streptomycin (50 µg/ml), and 7.5% FBS in 150 cm^2^ tissue culture flasks. The differentiation to monocytes/macrophages was initiated by incubation of cells (3 × 10^5^/ml) with 0.3 ng/ml TPA for 3 days, and then the medium was removed. The cells were fed with fresh media without further addition of TPA. The cells were cultured for another 3 days. Cells at this time were characteristic of monocytes/macrophages [21, 22] and were harvested for further experiments.

### Isolation of mitochondria from monocytes/macrophages

Mitochondria were isolated from the freshly harvested monocytes/macrophages according to the method of Rickwood et al. [23] with minor modifications. Briefly, cells (4–6 × 10^7^ cells per sample) were washed once with PBS. The cell pellet was resuspended in 5 ml sucrose buffer (0.25 M sucrose, 10 mM Hepes, 1 mM EGTA and 0.5% BSA, pH 7.4), homogenized in a Dounce tissue grinder on ice. The homogenate was centrifuged at 1,500 x *g* for 10 min at 4 °C. The supernatant was collected and centrifuged at 10,000 x *g* for 10 min at 4 °C. The resulting mitochondrial pellet was washed twice with 5 ml sucrose buffer and then resuspended in 1 ml sucrose buffer. The mitochondrial protein was measured with Bio-Rad protein assay dye based on the method of Bradford [24] with BSA as the standard.

### Measurement of mitochondrial O_2_ consumption

The rate of mitochondrial O_2_ consumption was measured polarographically with a Clark-type oxygen electrode (YSI-53, Yellow Springs, OH) at 37 °C in 2.5 ml of reaction mixture. The reaction mixture contained 0.5 mg protein/ml of mitochondria in the presence or absence of exogenous substrates in respiration buffer (70 mM sucrose, 220 mM mannitol, 2 mM Hepes, 2.5 mM KH_2_PO_4_, 2.5 mM MgCl_2_, 0.5 mM EDTA, and 0.1% BSA, pH 7.4). The mitochondrial substrates used included pyruvate/malate (6 mM/6 mM) or succinate (6 mM). To determine the redox cycling of BP-derived quinones, the stimulation of KCN-resistant mitochondrial O_2_ consumption was monitored following incubation of the substrate-supported mitochondria with various BP-derived quinones in the presence of 0.2 mM KCN at 37 °C.

### Detection of intramitochondrial superoxide (O_2_⋅^−^) and extramitochondrial H_2_O_2_ production

Lucigenin- and luminol-derived chemiluminescence (CL) assays were used to detect intramitochondrial O_2_⋅^−^ and extramitochondrial H_2_O_2_ production, respectively [25]. In brief, the reaction mixture contained 0.5 mg protein of mitochondria in 1 ml respiration buffer in the presence or absence of exogenous substrates or other agents. The mitochondria substrates used for CL experiments included pyruvate/malate (6 mM/6 mM), succinate (6 mM) or ascorbate/TMPD (1 mM/0.2 mM). Ascorbate and TMPD are artificial substrates that can directly feed electrons to complex IV, leading to mitochondrial O_2_ consumption [26]. The lucigenin-derived CL response was initiated by adding 5 µM lucigenin to the reaction mixture. To initiate the luminol-derived CL, 5 µM luminol and 10 µg/ml HRP were added to the reaction mixture. The CL was continuously measured with a Berthod LB9505 six-channel luminometer (Wildbad, Germany) at 37 °C for 60 min. Data from CL experiments are expressed as integrated area under the curve.

### Electron paramagnetic resonance (EPR) measurement of mitochondrial O_2_⋅^−^ production

DEPMPO spin trapping was used to measure O_2 _⋅^**−**^ production by isolated mitochondria. Briefly, mitochondria (0.5 mg protein) were incubated with 10 mM DEPMPO in the presence of exogenous added substrates plus other agents in 0.5 ml respiration buffer at 37 °C. The mitochondrial substrates used for spin trapping experiments included pyruvate/malate (6 mM/6 mM) and succinate (6 mM). After 15 min incubation, the sample was immediately transferred to an EPR flat cell. The EPR spectra were recorded at room temperature with a Bruker EPR spectrometer operating at X-band as described before [27, 28].

### Statistical analysis

Data are presented as mean ± S.E. from at least three independent experiments unless otherwise indicated. Comparisons between two groups were conducted using a two-tailed Student’s t-test. Comparisons among multiple groups were performed using one-way analysis of variance (ANOVA) followed by Tukey’s multiple-comparison post hoc test. A value of p *<* 0.05 was considered statistically significant.

## Results and discussion

### BP-derived quinones undergo active mitochondrial redox cycling

**Fig. 2:**
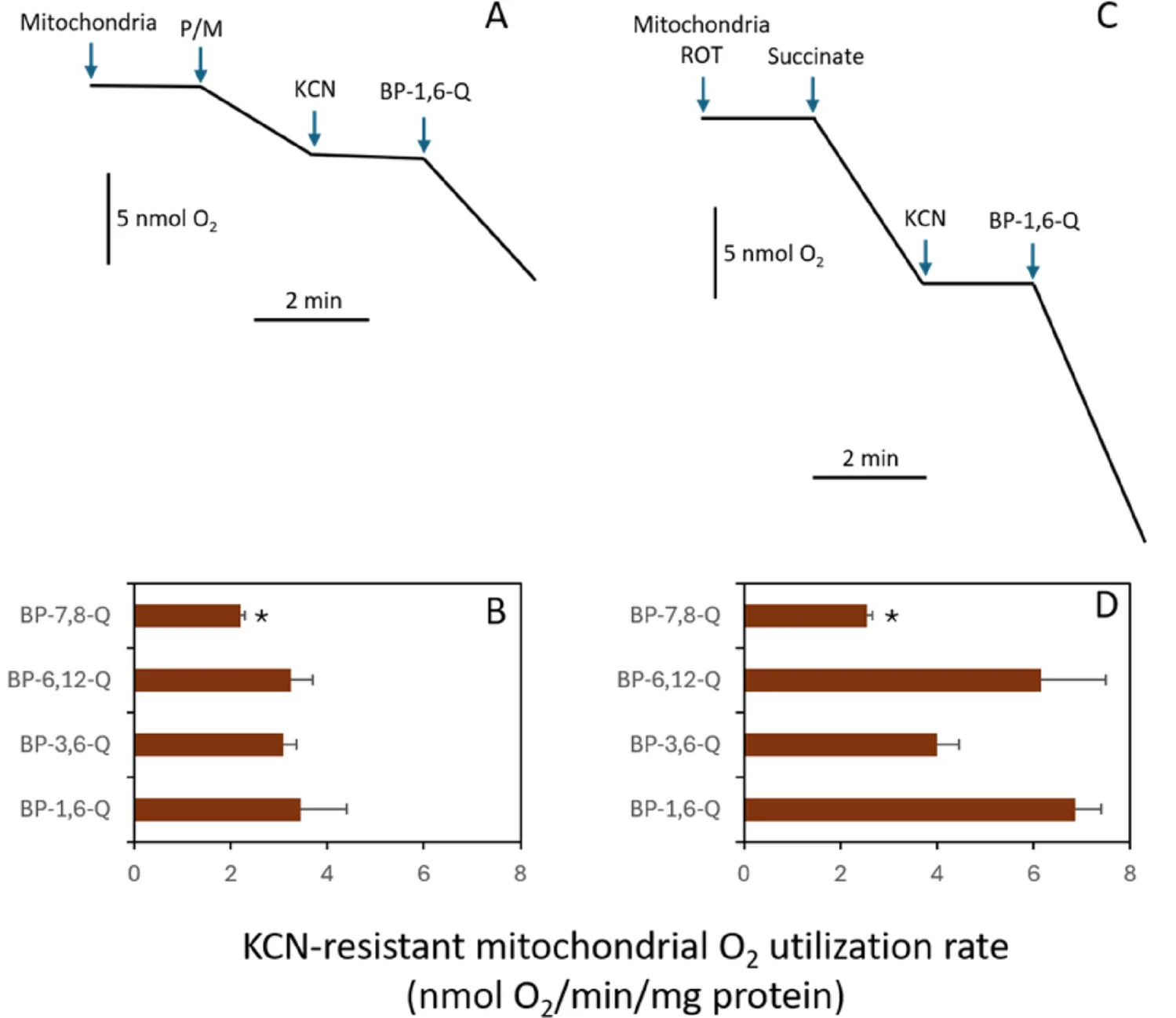
Stimulation of KCN-resistant O_2_ consumption by BP-derived quinones with isolated mitochondria. The KCN-resistant O_2_ consumption was detected following incubation of the pyruvate/malate (panels **A** and **B**)- or succinate (panels **C** and **D**)-supported mitochondria with the indicated BP-derived quinones at 10 µM, as described under Materials and Methods. In panels **A** and **C**, the sequence of addition of the various agents to the mitochondria was indicated. Data in panels **A** and **C** represent mean of three independent experiments, each performed in triplicate. Data in panels B and D represent mean ± S.E. of three independent experiments, each performed in triplicate. *, significantly different from BP-1,6-Q, BP-3,6-Q, or BP-6,12-Q

Quinone redox cycling involves one-electron reduction of quinones to semiquinone radicals followed by rapid reoxidation by O_2_, regenerating the parent quinone while producing ROS [13, 14]. To determine whether BP-derived quinones undergo mitochondrial redox cycling, KCN-resistant O_2_ consumption was measured in isolated mitochondria energized with either pyruvate/malate or succinate. Under these conditions, KCN inhibits cytochrome c oxidase, thereby blocking normal respiratory O_2_ consumption and allowing O_2_ utilization to serve as an indicator of quinone-dependent redox cycling. All four BP-derived quinones stimulated marked KCN-resistant O_2_ consumption (Fig. 2), indicating that they readily undergo catalytic redox cycling within intact mitochondria. BP-1,6-Q, BP-3,6-Q, and BP-6,12-Q consistently exhibited greater activity than BP-7,8-Q, while BP-1,6-Q and BP-6,12-Q produced higher rates of O_2_ consumption in succinate-supported than in pyruvate/malate-supported mitochondria. These findings suggest that both quinone structure and the respiratory substrate influence the efficiency of mitochondrial redox cycling of BP-derived quinones. Nevertheless, our results clearly show that intact mitochondria efficiently support the catalytic redox cycling of several major BP-derived quinones. Rather than functioning solely as electrophilic metabolites, these quinones appear to continuously exploit reducing equivalents generated during oxidative phosphorylation, suggesting mitochondria as an active catalytic platform for BP quinone bioactivation. It should be noted that, because of the limited sensitivity of oxygen polarography, relatively high micromolar concentrations of BP quinones were required to assess their capacity to undergo redox cycling.

### BP-derived quinones stimulate mitochondrial ROS production

**Fig. 3:**
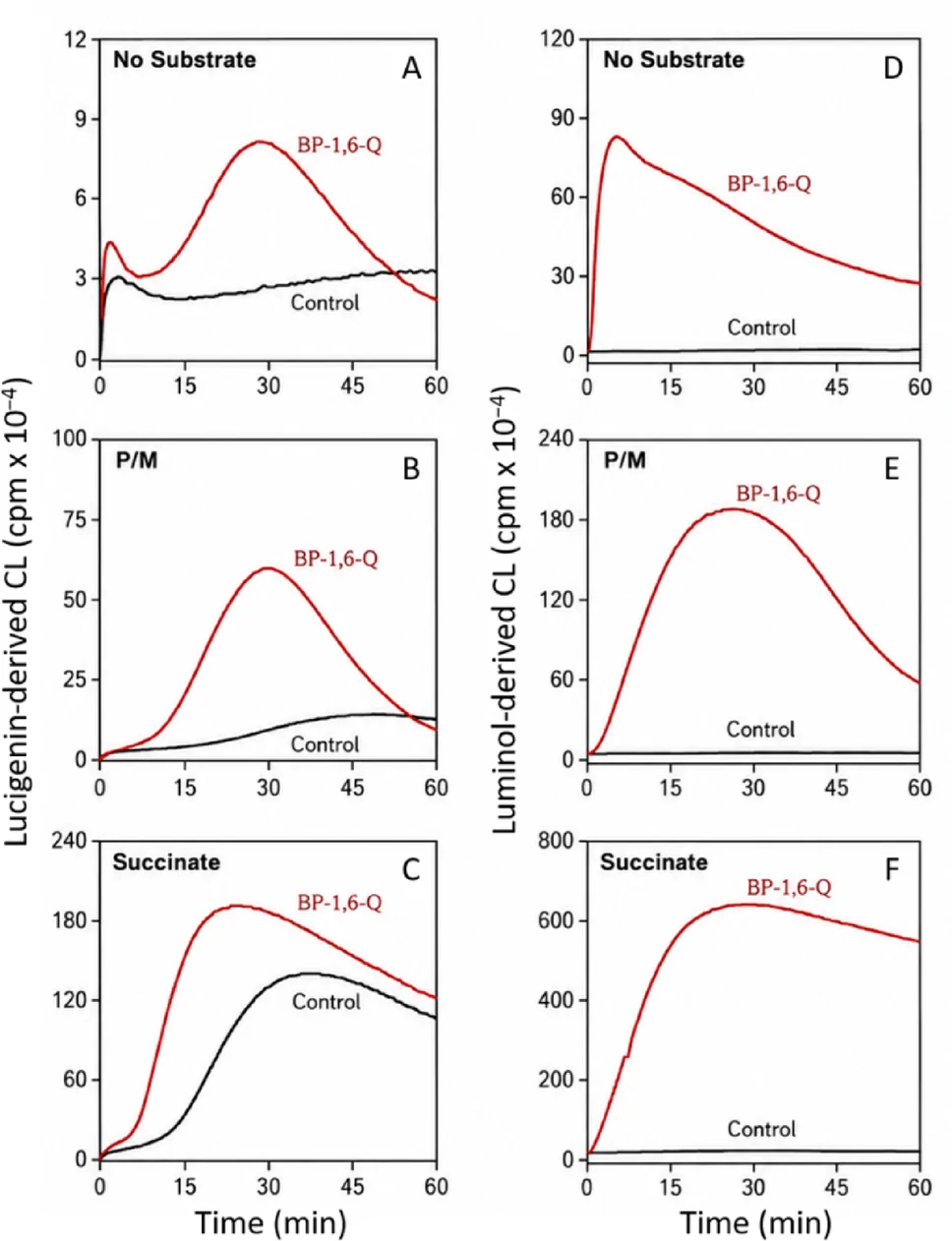
Stimulation of mitochondrial ROS production by BP-1,6-Q with isolated mitochondria. The mitochondria were incubated with 1 µM BP-1,6-Q in the presence or absence of pyruvate/malate or succinate. Production of mitochondrial ROS was detected by lucigenin (panels **A**, **B** and **C**)- and luminol (panels **D**, **E** and **F**)-derived CL, as described under Materials and Methods. The representative CL curves of at least three independent experiments are shown. Control denotes the ROS production in the absence of BP-1,6-Q

Because quinone redox cycling inevitably generates ROS, we next examined mitochondrial O_2_⋅^**−**^ and H_2_O_2_ production using lucigenin- and luminol-derived CL, respectively [25]. BP-1,6-Q markedly increased both lucigenin- and luminol-derived CL (Fig. 3), suggesting enhanced production of intramitochondrial O_2_⋅^−^ and extramitochondrial H_2_O_2_. Although BP-1,6-Q stimulated ROS production in the absence of added respiratory substrates, the response increased substantially in mitochondria energized with either pyruvate/malate or succinate, indicating that active electron transport markedly enhances BP quinone redox cycling. This substrate dependence provides important mechanistic insight. Had ROS generation occurred independently of the respiratory chain, substrate addition would have produced little enhancement. Instead, the marked stimulation observed in energized mitochondria indicates that BP-derived quinones intercept electrons from the METC, thereby converting physiological electron flow into sustained ROS production. Thus, mitochondrial respiration is not merely associated with quinone redox cycling but actively fuels the catalytic process.

### Mitochondria efficiently amplify BP quinone-induced oxidative stress

**Fig. 4:**
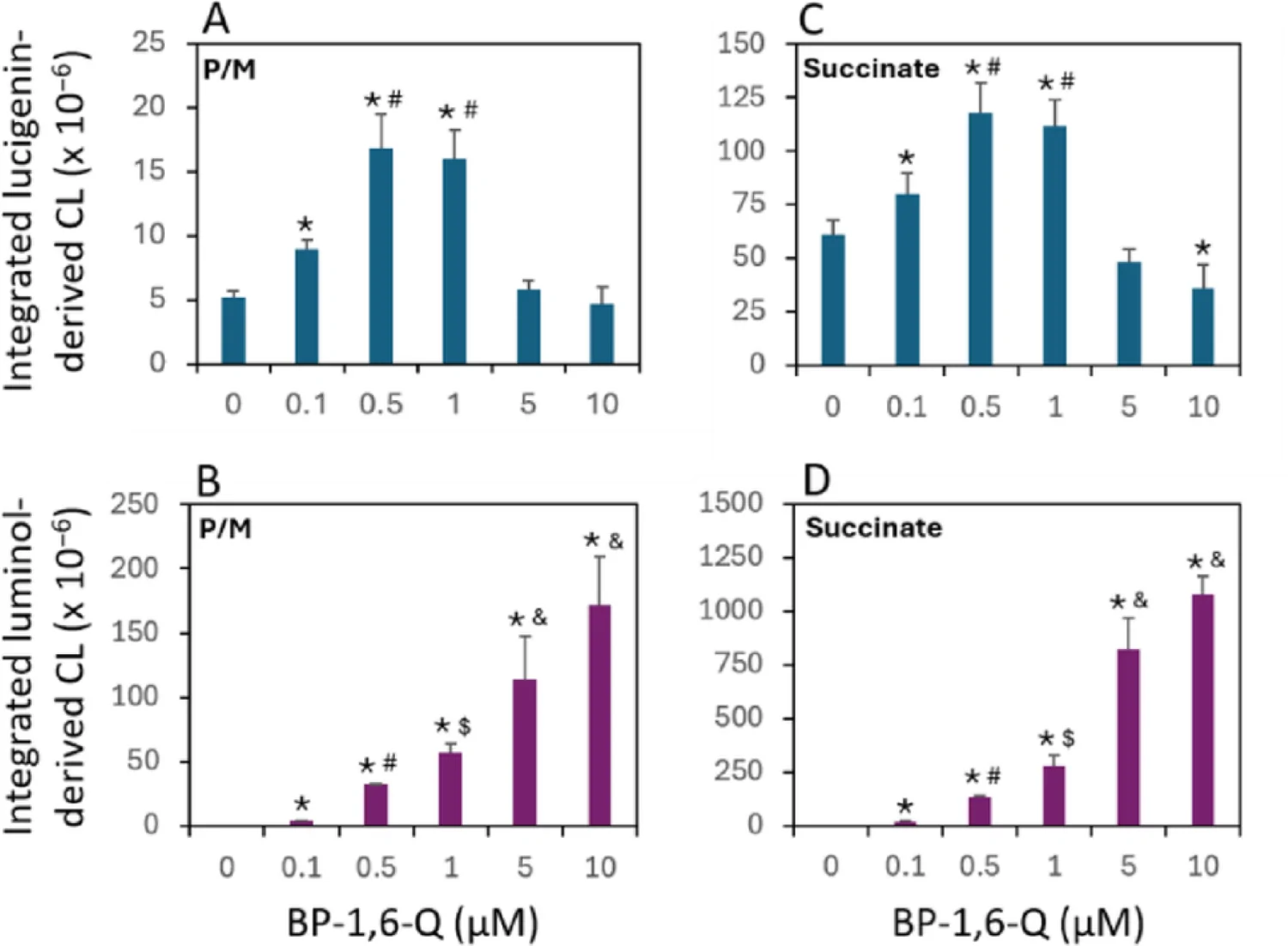
Concentration-dependent stimulation of mitochondrial ROS production by BP-1,6-Q with isolated mitochondria. The mitochondria were incubated with the indicated concentrations of BP-1,6-Q in the presence of pyruvate/malate (panels **A** and **B**) or succinate (panels **C** and **D**). Production of mitochondrial ROS was detected by lucigenin (panels **A** and **C**)- and luminol (panels **B** and **D**)-derived CL, as described under Materials and Methods. Data represent mean ± S.E. of at least three independent experiments, each performed in triplicate. *, significantly different from 0 µM BP-1,6-Q; #, significantly different from 0.1 µM BP-1,6-Q; $, significantly different from 0.5 µM BP-1,6-Q; &, significantly different from 1 µM BP-1,6-Q

To evaluate the efficiency of mitochondrial ROS stimulation, isolated mitochondria were incubated with increasing concentrations of BP-1,6-Q. Both O_2 _⋅^**−**^ and H_2_O_2_ production increased significantly at concentrations as low as 0.1 µM of BP-1,6-Q (Fig. 4). H_2_O_2_ production increased progressively throughout the concentration range examined, whereas lucigenin-derived CL reached a maximum at lower BP-1,6-Q concentrations before declining at higher concentrations. The reduction in lucigenin-derived CL at higher BP-1,6-Q concentrations is unlikely to reflect diminished O_2 _⋅^**−**^ production because H_2_O_2_ generation continued to increase. Instead, previous studies demonstrated that lucigenin accumulation depends on preservation of the mitochondrial membrane potential [29]. High concentrations of BP-1,6-Q likely impair mitochondrial bioenergetics, reducing membrane potential and consequently decreasing intramitochondrial lucigenin accumulation despite continued ROS production. Perhaps the most significant observation is the remarkable efficiency with which mitochondria amplify BP quinone-mediated ROS production. Nanomolar-to-submicromolar concentrations of BP-1,6-Q produced readily detectable ROS, indicating that continuous catalytic cycling within the METC greatly magnifies the oxidative potential of BP-derived quinones. Unlike conventional oxidants that are consumed during reactions, BP quinones are regenerated after each catalytic cycle, allowing repeated transfer of electrons from mitochondrial respiratory components to O_2_. Consequently, even relatively low intracellular BP quinone concentrations can sustain prolonged ROS production.

### Structural differences among BP-derived quinones influence mitochondrial ROS generation

**Fig. 5:**
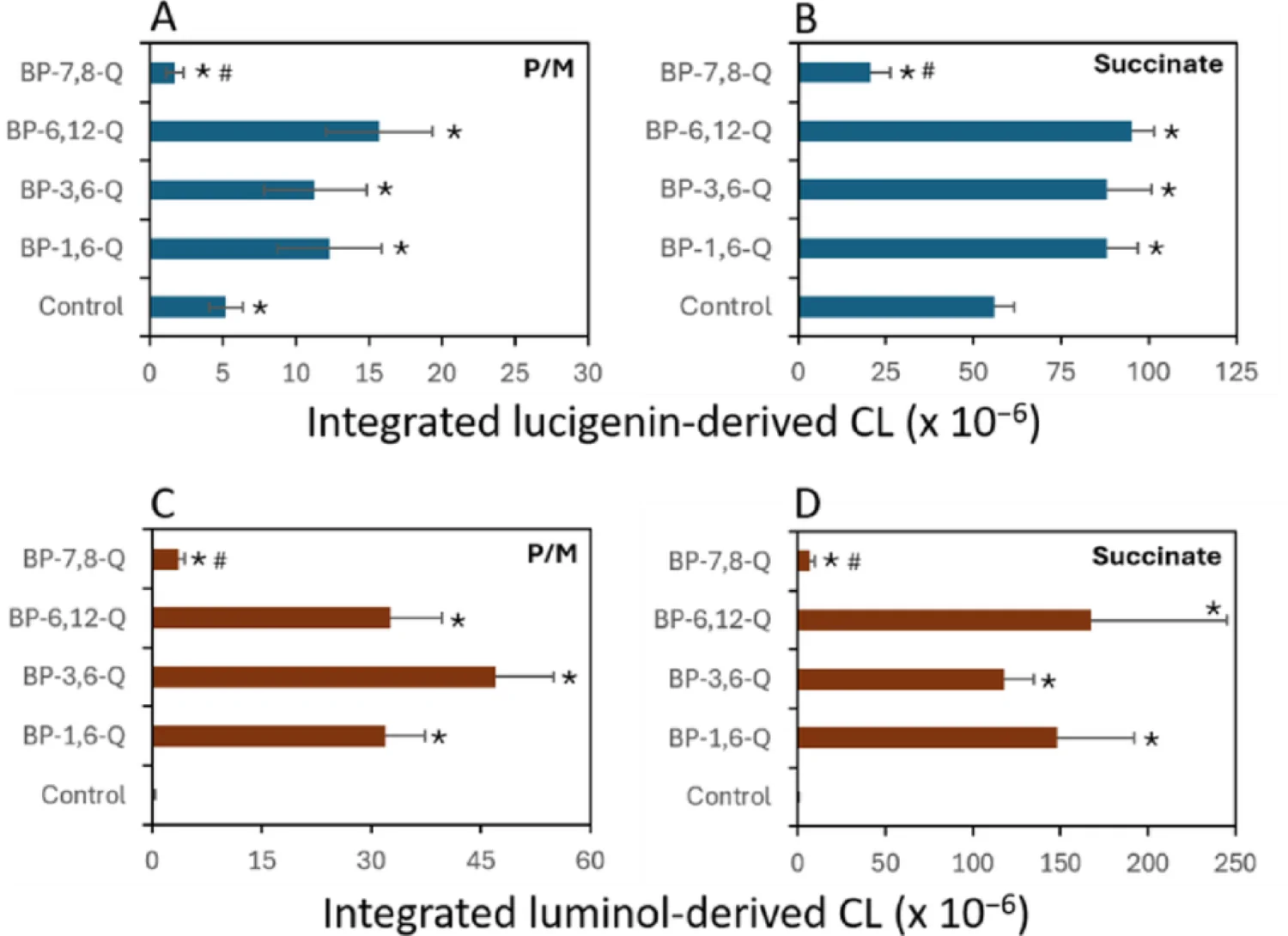
Induction of mitochondrial ROS production by different BP-derived quinones with isolated mitochondria. The mitochondria were incubated with the indicated BP-derived quinones at 1µM in the presence of pyruvate/malate (panels **A** and **C**) or succinate (panels **B** and **D**). Production of mitochondrial ROS was detected by lucigenin (panels **A** and **B**)- and luminol (panels **C** and **D**)-derived CL, as described under Materials and Methods. Data represent mean ± S.E. of at least three independent experiments, each performed in triplicate. *, significantly different from Control. #, significantly different from BP-1,6-Q, BP-3,6-Q, or BP-6,12-Q

The four BP-derived quinones examined differed substantially in their ability to stimulate mitochondrial ROS production (Fig. 5). BP-1,6-Q, BP-3,6-Q, and BP-6,12-Q all produced robust increases in both O_2_⋅^**−**^ and H_2_O_2_ formation, whereas BP-7,8-Q exhibited substantially weaker activity and, under several conditions, reduced lucigenin-derived CL despite increasing H_2_O_2_ production. These findings indicate that relatively subtle structural differences among BP-derived quinones markedly influence their mitochondrial redox behavior. The reduced activity of BP-7,8-Q is unlikely to reflect an inability to undergo redox cycling because H_2_O_2_ production remained elevated. Instead, BP-7,8-Q may exert additional electrophilic effects on mitochondrial function. Previous studies demonstrated that BP-7,8-Q readily reacts with sulfhydryl groups [17], and modification of critical mitochondrial thiols could impair electron transport and decrease membrane potential, thereby reducing lucigenin accumulation while allowing continued H_2_O_2_ release. Collectively, these findings suggest that BP-derived quinones are not functionally equivalent. Rather, their ability to undergo mitochondrial redox cycling and generate ROS is determined not only by their redox properties but also by their interactions with specific mitochondrial targets. Although directly comparable redox potentials are not available for all BP quinones under physiological conditions, BP quinones readily undergo one-electron reduction to semiquinone intermediates and can serve as electron acceptors for NAD(P)H-dependent oxidoreductases [16]. Their redox properties are therefore compatible with electron acquisition from reduced centers within complexes I–III, although the contribution of each site is also governed by quinone accessibility, binding, and electron-transfer kinetics rather than redox potential alone.

### EPR spin trapping confirms mitochondrial O_2_.^−^ generation during BP quinone redox cycling

**Fig. 6:**
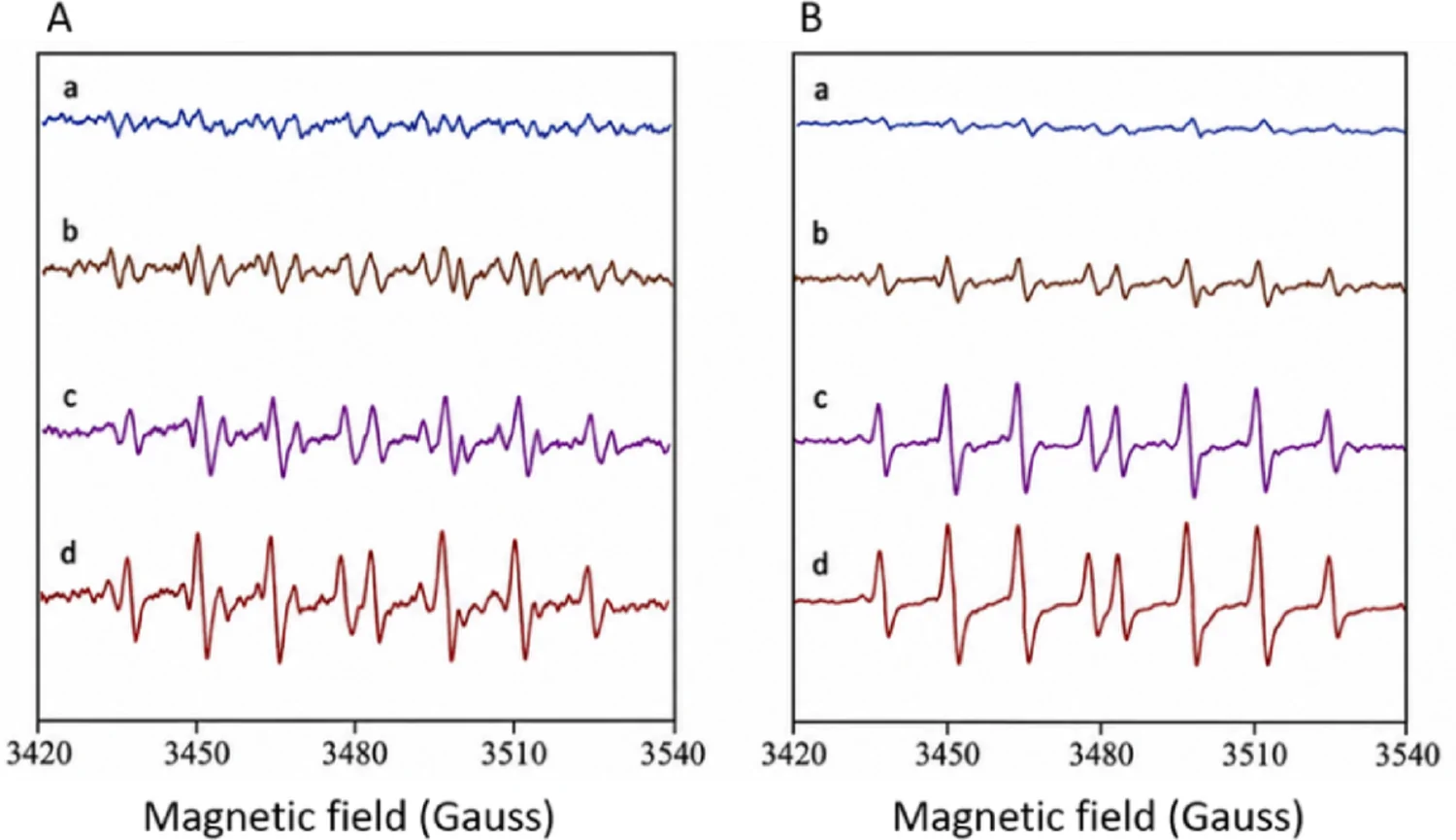
Formation of DEPMPO-OH adduct with isolated mitochondria following treatment with BP-1,6-Q. The mitochondria were incubated with 10 mM DEPMPO and various concentrations of BP-1,6-Q in the presence of pyruvate/malate (panel **A**) or succinate (panel **B**). Formation of the DEPMPO spin adduct (DEPMPO-OH) was detected by EPR spectroscopy as described under Materials and Methods. In both panels **A** and **B**, spectra a, b, c, and d represent control, 0.1 µM BP-1,6-Q, 1 µM BP-1,6-Q, and 2.5 µM BP-1,6-Q, respectively. The representative EPR spectra of at least three independent experiments are shown

**Fig. 7:**
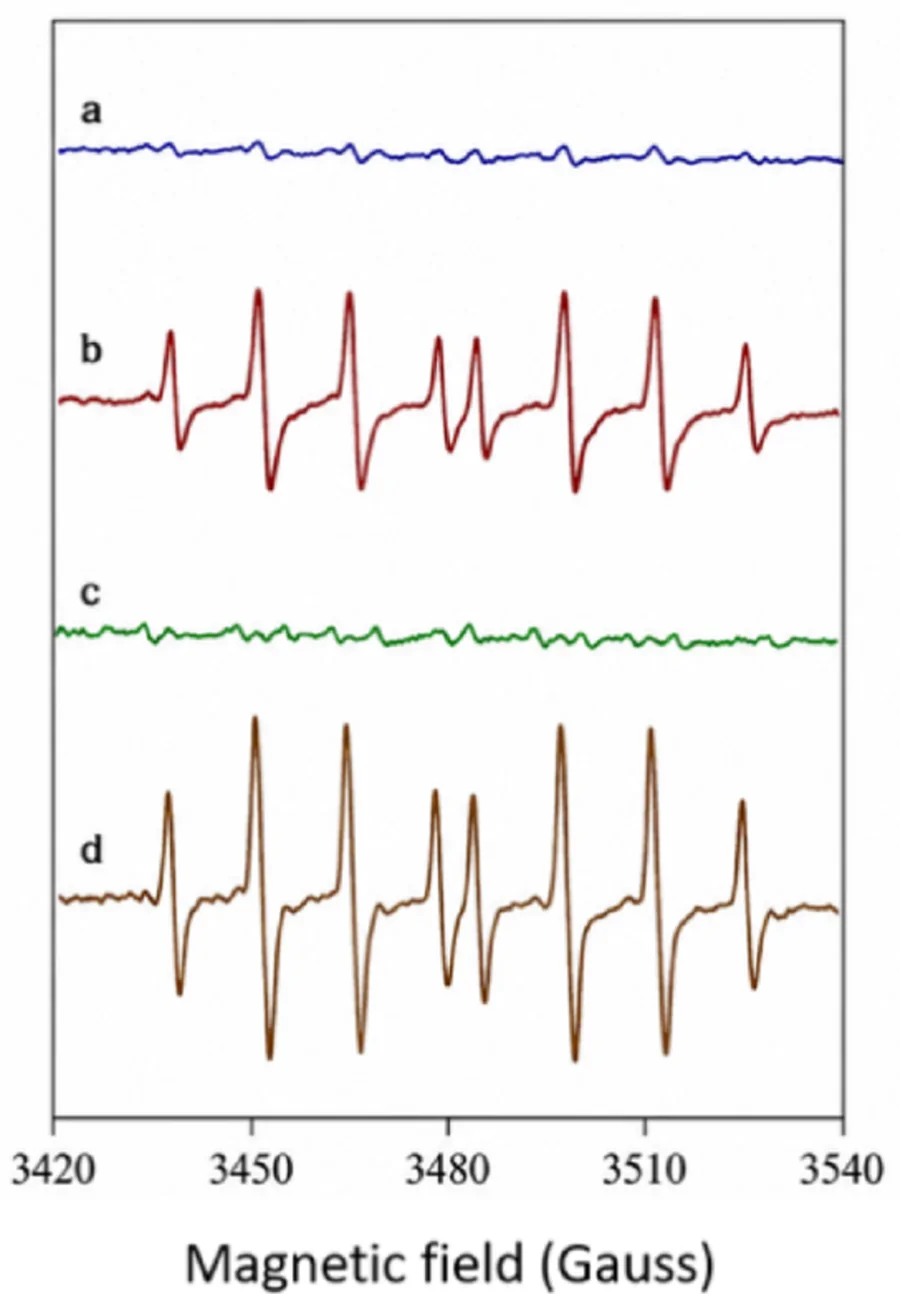
Effects of Cu, Zn SOD and catalase on BP-1,6-Q-induced formation of DEPMPO-OH adduct with isolated mitochondria. The mitochondria were incubated with succinate, 10 mM DEPMPO and 2.5 µM BP-1,6-Q in the presence or absence of 500 units/ml of Cu, Zn SOD or catalase. Formation of the DEPMPO-OH adduct was detected by EPR spectroscopy as described under Materials and Methods. Spectra **a**, **b**, **c**, and **d** represent control, BP-1,6-Q, BP-1,6-Q plus Cu, Zn SOD, and BP-1,6-Q plus catalase, respectively. The representative EPR spectra of at least three independent experiments are shown

To independently verify that BP quinone redox cycling generates O_2_⋅^**−**^, EPR spectroscopy using the spin trap DEPMPO was employed. BP-1,6-Q induced concentration-dependent formation of the characteristic DEPMPO-OH adduct in both pyruvate/malate- and succinate-supported mitochondria (Fig. 6). Consistent with the CL studies, radical formation was substantially greater in succinate-supported mitochondria, indicating that electron flow through the FAD-linked respiratory pathway more efficiently supports BP quinone reduction. Although the predominant spin adduct detected was DEPMPO-OH, previous studies demonstrated that the initially formed DEPMPO-superoxide adduct can be rapidly converted into the more stable hydroxyl adduct [27, 28]. Thus, formation of DEPMPO-OH is widely accepted as evidence of O_2_⋅^−^ generation under these conditions. The identity of the radical species was further examined using Cu, Zn SOD and catalase (Fig. 7). Cu, Zn SOD completely abolished DEPMPO-OH formation, confirming that O_2_⋅^−^ is the primary ROS generated during BP quinone redox cycling. Because exogenous Cu, Zn SOD cannot readily cross the inner mitochondrial membrane, this observation also indicates that a substantial fraction of mitochondrial O_2_⋅^−^ escapes into the extramitochondrial space. Notably, catalase enhanced rather than diminished DEPMPO-OH formation. Although the mechanism remains unclear, removal of H_2_O_2_ by catalase may shift the steady-state equilibrium toward continued O_2_.^−^ generation. Regardless of the underlying mechanism, these findings confirm that O_2_⋅^−^ is the primary radical produced during mitochondrial BP quinone redox cycling.

Taken together, the experiments described above support three notable conclusions. First, BP-derived quinones are highly efficient substrates for mitochondrial redox cycling. Second, active electron transport markedly amplifies BP quinone-dependent ROS production. Third, individual BP quinones differ substantially in their redox-cycling efficiencies, indicating selective interactions with components of the respiratory chain. These findings prompted the next critical question: which mitochondrial respiratory complexes donate electrons to BP-derived quinones? To address this question, as described next, we used selective inhibitors of individual respiratory complexes to identify the sites responsible for BP quinone reduction and ROS generation. Although all four BP quinones were screened to establish the generality of mitochondrial ROS amplification, BP-1,6-Q was subsequently used as a representative one for mechanistic studies to determine the METC sites supporting BP quinone redox cycling.

### Mitochondrial complex I is a major catalytic site for BP quinone redox cycling

**Fig. 8:**
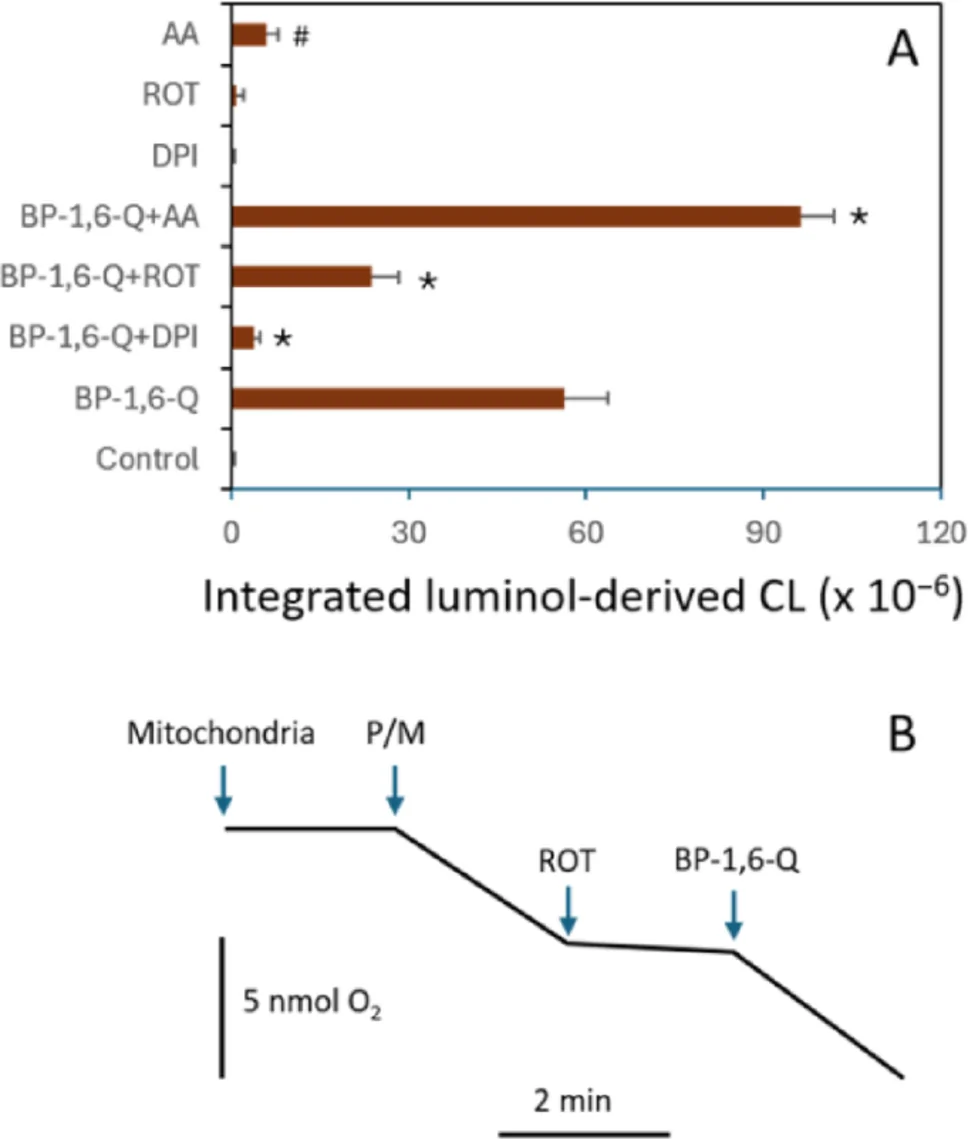
Involvement of complex I in mediating the redox cycling of BP-1,6-Q with isolated mitochondria. In panel **A**, the mitochondria were incubated with pyruvate/malate (P/M) and 1 µM BP-1,6-Q in the presence or absence of DPI (10 µM), ROT (10 µM), or AA (1 µM). The mitochondrial H_2_O_2_ production was detected by luminol-derived CL. In panel **B**, the mitochondria were incubated with pyruvate/malate, followed by the addition of 10 µM ROT and 10 µM BP-1,6-Q as indicated by the arrows. The mitochondrial O_2_ consumption was measured as described under Materials and Methods. Data in panel A represent mean ± S.E. of at least three independent experiments, each performed in triplicate. Curve in panel **B** represents mean from three experiments. *, significantly different from BP-1,6-Q alone; #, significantly different from Control

Complex I is the primary entry point for NADH-derived electrons into the METC and has previously been implicated in the redox cycling of xenobiotics such as doxorubicin [30]. We therefore investigated the contribution of complex I to BP quinone redox cycling using the selective inhibitors ROT and DPI. ROT inhibits electron transfer from the terminal iron-sulfur cluster (N2) to ubiquinone, whereas DPI blocks electron flow at the FMN-containing NADH dehydrogenase domain by preventing the initial oxidation of NADH ([31, 32]. In pyruvate/malate-supported mitochondria, ROT reduced BP-1,6-Q-induced H₂O₂ production by approximately 60%, whereas DPI almost completely abolished the response (Fig. 8A). In addition, BP-1,6-Q stimulated substantial ROT-resistant O_2_ consumption (Fig. 8B). ROT blocks electron transfer from complex I to ubiquinone, thereby preventing oxygen consumption at complex IV and allowing oxygen consumption specifically attributable to BP-1,6-Q redox cycling to be measured. Together, these findings strongly implicate the NADH dehydrogenase region of complex I in mediating BP quinone redox cycling.

The differential effects of ROT and DPI also provide mechanistic insight. Whereas DPI completely blocked BP quinone redox cycling and ROS production by preventing electron entry into complex I, ROT only partially inhibited the redox cycling and ROS production, suggesting that BP quinones intercept electrons both upstream and downstream of the ROT-sensitive step. These observations support a model in which BP quinone redox cycling occurs at two potential sites within complex I: (1) the FMN/early iron-sulfur cluster region, which is also recognized as a major site of endogenous O_2_.^−^ generation by complex I [33], and (2) the ubiquinone-binding region near the terminal N2 iron-sulfur cluster. Owing to its highly lipophilic nature, BP-1,6-Q may diffuse into the hydrophobic ubiquinone-binding pocket and compete with endogenous ubiquinone for electrons destined for physiological electron transport. Complete inhibition by DPI is consistent with this model, as blockade of NADH oxidation eliminates electron flow through complex I and thereby prevents BP quinone reduction at both proposed sites. Conversely, the enhancement of BP-1,6-Q-induced H₂O₂ production by AA in pyruvate/malate-driven mitochondria (Fig. 8A) is consistent with increased accumulation of electrons upstream of the antimycin-sensitive site. The resulting increase in electron availability within complex I would be expected to enhance electron transfer to BP-1,6-Q, thereby promoting BP quinone redox cycling and ROS production.

### Complex III provides another catalytic site supporting BP quinone redox cycling

**Fig. 9:**
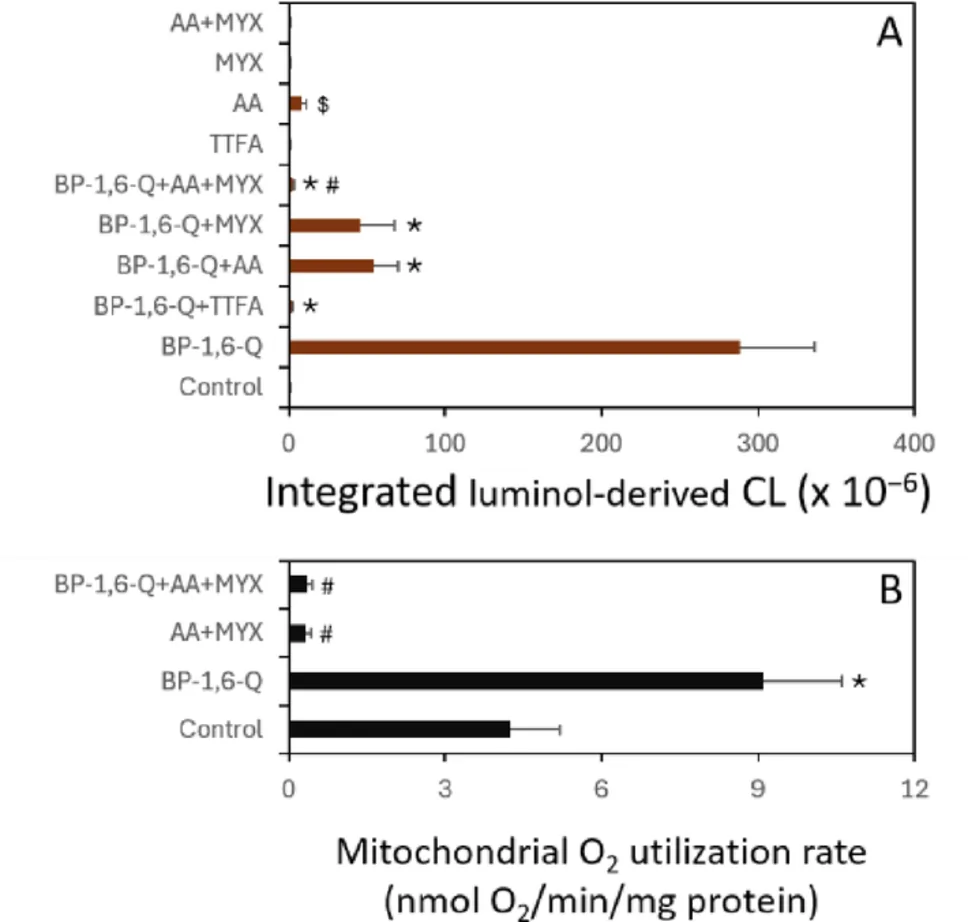
Involvement of complex III in mediating the redox cycling of BP-1,6-Q with isolated mitochondria. In panel **A**, the mitochondria were incubated with succinate, 10 µM ROT, and 1 µM BP-1,6-Q in the presence or absence of TTFA (0.2 mM), AA (20 µM), MYX (20 µM), or MYX plus AA (20 µM plus 20 µM). The mitochondrial H_2_O_2_ production was detected by luminol-derived CL, as described under Materials and Methods. In panel **B**, mitochondrial O_2_ consumption was measured following incubation of the succinate-supported mitochondria with 10 µM ROT and 10 µM BP-1,6-Q in the presence or absence of AA (20 µM) plus MYX (20 µM), as described under Materials and Methods. In both panels A and B, data represent mean ± S.E. of at least three independent experiments, each performed in triplicate. In panel **A**, *, significantly different from BP-1,6-Q alone; #, significantly different from BP-1,6-Q + AA, or BP-1,6-Q + MYX. In panel **B**, *, significantly different from Control; #, significantly different from BP-1,6-Q

Under physiological condition, in addition to NADH, succinate also feeds electrons to the METC through succinate dehydrogenase. We thus use succinate-driven mitochondria to determine the potential involvement of other respiratory complexes in BP quinone redox cycling. In succinate-supported mitochondria, inhibition of electron transfer from complex II to the ubiquinone pool by TTFA completely abolished BP-1,6-Q-induced H_2_O_2_ production (Fig. 9A). Since TTFA does not inhibit the catalytic center of succinate dehydrogenase itself, the above observation suggests that the catalytic center of succinate dehydrogenase is unlikely to contribute to BP-1,6-Q redox cycling. This contrasts with the findings for complex I, where blockade of electron transfer from NADH dehydrogenase to ubiquinone by ROT only partially inhibited BP-1,6-Q redox cycling (Fig. 8A). These results implicate that the catalytic center of complex II is not the site of BP quinone reduction but instead supplies electrons to downstream respiratory components, such as complexes III and IV [31]. In this context, both AA and MYX markedly inhibited BP-1,6-Q-induced H_2_O_2_ production, while combined treatment with both inhibitors almost completely abolished H_2_O_2_ generation and KCN-resistant O_2_ consumption (Fig. 9). Because these inhibitors block opposite sides of the Q cycle in complex III [34], their combined effect suggests that continuous electron flow through complex III is required to sustain BP quinone redox cycling. Notably, the apparent site responsible for BP quinone reduction differs from that responsible for endogenous complex III-derived O_2_.^**−**^ production. Endogenous O_2_.^**−**^ originates primarily from ubisemiquinone intermediates proximal to the AA-sensitive site [25, 35], whereas the present inhibitor profile suggests that BP quinones interact with distinct electron-transfer components within the Q cycle. Although the precise electron donor remains to be identified, these findings clearly indicate complex III as another major site supporting BP quinone redox cycling within the METC.

### Complex IV does not participate in BP quinone redox cycling

**Fig. 10:**
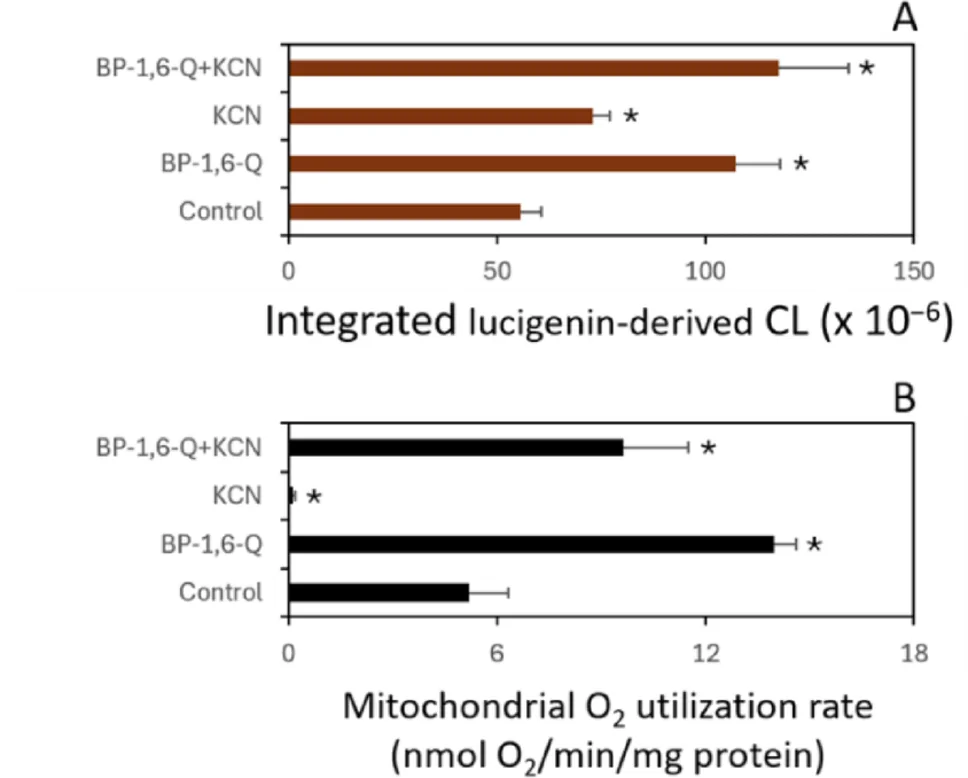
Involvement of complex IV in mediating the redox cycling of BP-1,6-Q with isolated mitochondria. Lucigenin-derived CL (panel **A**) and mitochondrial O_2_ consumption (panel **B**) were measured following incubation of the succinate-supported mitochondria with BP-1,6-Q at 1 µM (panel **A**) or 10 µM (panel **B**) in the presence or absence of 0.2 mM KCN, as described under Materials and Methods. Data in both panels **A** and **B** represent mean ± S.E. of at least three independent experiments, each performed in triplicate. *, significantly different from Control

Complex IV catalyzes the terminal four-electron reduction of O_2_ to water and therefore represents the final electron acceptor of the METC. To determine whether it contributes to BP quinone redox cycling, mitochondria were energized with ascorbate/TMPD, which donates electrons directly to cytochrome c and complex IV. Mitochondria supported by ascorbate/TMPD did not result in any significant production of either lucigenin- or luminol-derived CL (Data are not shown because the CL responses were indistinguishable from instrument background), which is consistent with the notion that complex IV is not involved in the reduction of O_2_ to form O_2_⋅^**−**^ and H_2_O_2_ [36]. Likewise, addition of BP-1,6-Q to the TMPD/ascorbate-supported mitochondria failed to stimulate any significant production of ROS as detected by luminol- and lucigenin-derived CL (Data are not shown because the CL responses were indistinguishable from instrument background). Because complex IV activity can be inhibited by KCN, we further examined the effects of KCN on the redox cycling of BP-1,6-Q with succinate-supported mitochondria. KCN inhibits peroxidases, thereby eliminating the use of the luminol/HRP system to measure the H_2_O_2_ formation by mitochondria. Lucigenin-derived CL was thus used to detect the intramitochondrial O_2_⋅^**−**^ production. As shown in Fig. 10A, inhibition of complex IV by KCN with succinate-driven mitochondria did not diminish the BP-1,6-Q stimulated-lucigenin-derived CL. Although addition of KCN completely blocked the succinate-supported mitochondrial O_2_ utilization, the BP-1,6-Q-stimulated O_2_ consumption was not significantly affected by KCN (Fig. 10B). These complementary findings suggest that complex IV is unlikely to be a significant site of BP quinone redox cycling. Unlike complexes I and III, which contain single-electron redox intermediates capable of reducing exogenous quinones, complex IV catalyzes the highly regulated four-electron reduction of oxygen directly to water [36]. This catalytic mechanism appears incompatible with BP quinone redox cycling and explains why BP-derived quinones selectively exploit upstream respiratory complexes for one-electron reduction.

### Conclusions

**Fig. 11:**
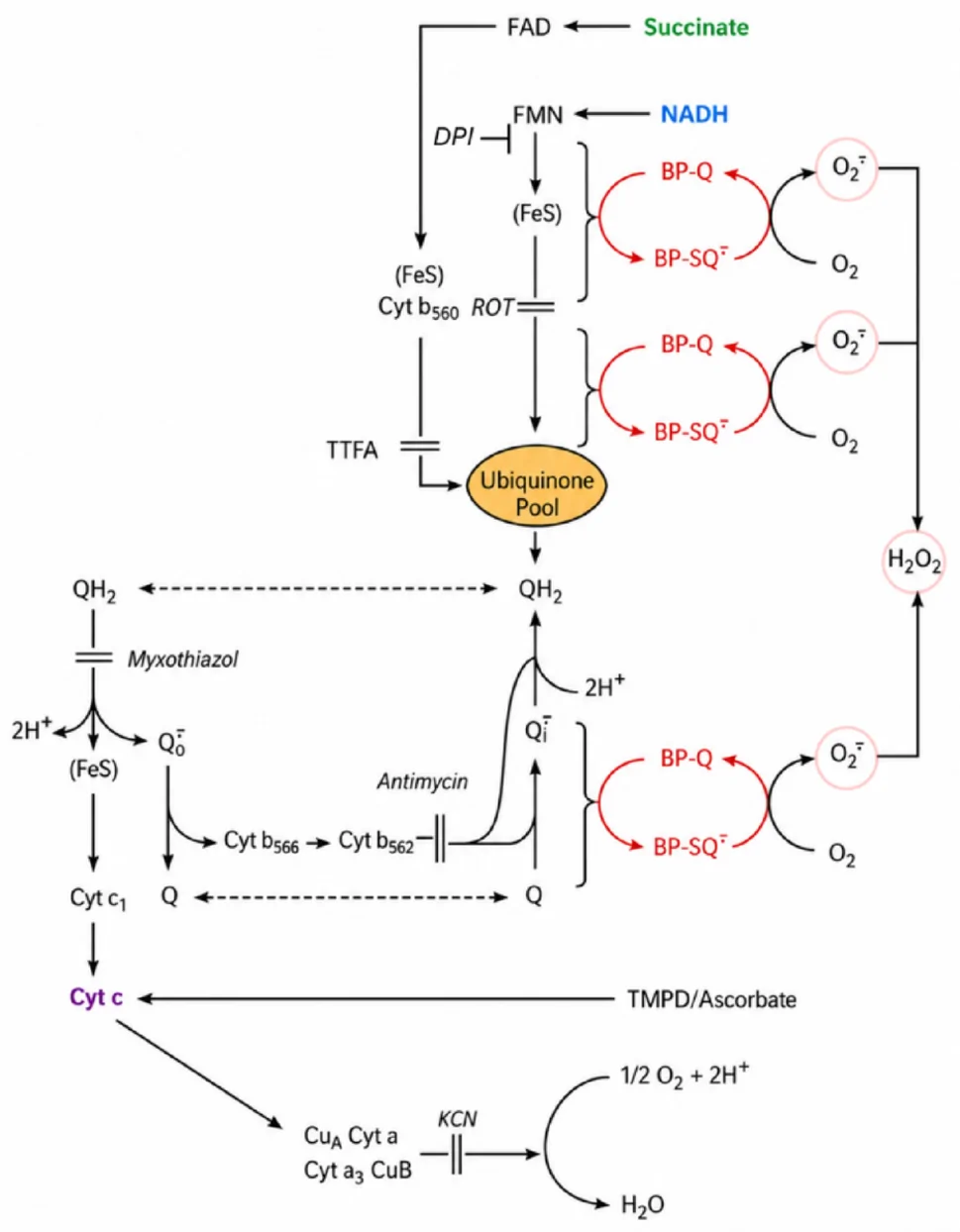
Proposed mechanism of mitochondrial BP quinone redox cycling. Electrons derived from NADH enter the METC through complex I, whereas electrons generated from succinate oxidation enter through complex II, and electrons from both pathways are transferred to the ubiquinone (Q) pool. Reduced ubiquinone (QH₂) donates electrons to complex III through the Q cycle, a process that transfers electrons to cytochrome c (Cyt c) en route to complex IV, where O_2_ is reduced to water. In this proposed model, BP-derived quinones (BP-Q) appear to undergo catalytic redox cycling by accepting electrons from the METC, primarily at complexes I and III. The inhibitor studies suggest that complex I may support BP quinone reduction at both the FMN/early Fe–S cluster region and the ubiquinone-binding site near the terminal N2 Fe–S cluster. In addition, complex III appears to represent a second major catalytic site, where BP quinone redox cycling likely occurs downstream of the inhibitory site of AA

Collectively, our experiments suggest complexes I and III as the principal catalytic sites supporting mitochondrial BP quinone redox cycling. Electrons derived from NADH or succinate are transferred through the respiratory chain, where BP-derived quinones intercept reducing equivalents primarily at complexes I and III (Fig. 11). One-electron reduction generates unstable semiquinone radicals that rapidly reduce O_2_, producing O_2_⋅^**−**^ while regenerating the parent BP quinone. Continuous repetition of this cycle allows a single BP quinone molecule to generate ROS without being consumed. This mechanism fundamentally distinguishes BP-derived quinones from conventional oxidants. Rather than acting stoichiometrically, BP quinones appear to function as catalytic electron shuttles, repeatedly diverting respiratory electrons from oxidative phosphorylation to O_2_, producing ROS. Consequently, mitochondria may serve not simply as passive targets of BP quinone toxicity but as highly efficient amplifiers of BP quinone-induced oxidative stress, which may. represent a potential contributor to BP toxicity.

It should be noted that this study is not without limitations. First, pharmacological inhibitors of the METC have inherent limitations. For example, DPI inhibits multiple flavoproteins in addition to complex I, whereas AA and MYX can alter endogenous mitochondrial ROS production, potentially complicating the interpretation of BP quinone-dependent ROS measurements. Therefore, the inhibitor data should be interpreted together with our complementary findings from isolated mitochondria and multiple inhibitor approaches, which collectively support the proposed involvement of complexes I and III in BP quinone redox cycling. Second, individual ROS assays also have inherent limitations, particularly the mitochondrial membrane potential-dependent accumulation of lucigenin and its potential redox-cycling properties at high concentrations [25, 28]. Accordingly, the use of complementary approaches for ROS detection, including lucigenin-derived CL, luminol-derived CL, EPR spin trapping, and KCN-resistant O_2_ polarography, was essential for reliably assessing BP quinone-induced ROS generation. Third, mitochondria isolated from human ML-1-derived monocytes/macrophages were used because the primary objective of this study was to elucidate the mitochondrial mechanism underlying BP quinone-induced dysfunction in the monocytic cells, a major research project of our laboratory. Although the METC is highly conserved among mammalian cells, the extent of BP quinone redox cycling and ROS generation may vary depending on mitochondrial maturity, metabolic state, and antioxidant capacity. Thus, while the underlying mechanism is likely to be applicable to other cell types, its quantitative contribution will require further experimental validation. Likewise, future studies are warranted to assess the pathophysiological consequences of METC-mediated BP quinone redox cycling and ROS production, including their impact on mitochondrial bioenergetics, cellular metabolism, and immune cell function.

## Data Availability

No datasets were generated or analysed during the current study.

## Abbreviations

AA: Antimycin A
BP: Benzo[a]pyrene
BP-1,6-Q: Benzo[a]pyrene-1,6-quinone
BP-3,6-Q: Benzo[a]pyrene-3,6-quinone
BP-6,12-Q: Benzo[a]pyrene-6,12-quinone
BP-7,8-Q: Benzo[a]pyrene-7,8-quinone
CL: Chemiluminescence
DEPMPO: 5-(Diethoxyphosphoryl)-5-methyl-1-pyrroline N-oxide
DEPMPO-OH: DEPMPO-hydroxyl radical adduct
DPI: Diphenyleneiodonium
EPR: Electron paramagnetic resonance
FAD: Flavin adenine dinucleotide
FMN: Flavin mononucleotide
HRP: Horseradish peroxidase
KCN: Potassium cyanide
METC: Mitochondrial electron transport chain
MYX: Myxothiazol
NADH: Reduced nicotinamide adenine dinucleotide
PAH: Polycyclic aromatic hydrocarbon
Q: Ubiquinone
QH_2_: Ubiquinol
ROS: Reactive oxygen species
SOD: Superoxide dismutase
TMPD: N,N,N′,N′-Tetramethyl-p-phenylenediamine
TTFA: Thenoyltrifluoroacetone

## References

[1] Lioy PL, Waldman JM, Greenberg A, Harkov R, Pietarinen C (1988) The total human environmental exposure study (THEES) to benzo(a)pyrene: comparison of the inhalation and food pathways. Arch Environ Health 43:304–312. 10.1080/00039896.1988.10545954 3415358

[2] Phillips DH (1999) Polycyclic aromatic hydrocarbons in the diet. Mutat Res 443:139–147. 10.1016/s1383-5742(99)00016-2 10415437

[3] Moorthy B, Chu C, Carlin DJ (2015) Polycyclic aromatic hydrocarbons: from metabolism to lung cancer. Toxicol Sci 145:5–15. 10.1093/toxsci/kfv040 PMC4408964 25911656

[4] Anselstetter V, Heimpel H (1986) Acute hematotoxicity of oral benzo(a)pyrene: the role of the Ah locus. Acta Haematol 76:217–223. 10.1159/000206059 3107308

[5] Legraverend C, Harrison DE, Ruscetti FW, Nebert DW (1983) Bone marrow toxicity induced by oral benzo[a]pyrene: protection resides at the level of the intestine and liver. Toxicol Appl Pharmacol 70:390–401. 10.1016/0041-008x(83)90157-6 6314600

[6] Hough JL, Baird MB, Sfeir GT, Pacini CS, Darrow D, Wheelock C (1993) Benzo(a)pyrene enhances atherosclerosis in White Carneau and Show Racer pigeons. Arterioscler Thromb 13:1721–1727. 10.1161/01.atv.13.12.1721 8241091

[7] Legraverend C, Guenthner TM, Nebert DW (1984) Importance of the route of administration for genetic differences in benzo[a]pyrene-induced in utero toxicity and teratogenicity. Teratology 29:35–47. 10.1002/tera.1420290106 6322374

[8] Cavalieri EL, Rogan EG, Cremonesi P, Devanesan PD (1988) Radical cations as precursors in the metabolic formation of quinones from benzo[a]pyrene and 6-fluorobenzo[a]pyrene. Fluoro substitution as a probe for one-electron oxidation in aromatic substrates. Biochem Pharmacol 37:2173–2182. 10.1016/0006-2952(88)90578-3 2837229

[9] Flowers-Geary L, Harvey RG, Penning TM (1995) Identification of benzo[a]pyrene-7,8-dione as an authentic metabolite of (+/-)-trans-7,8-dihydroxy-7,8-dihydrobenzo[a]pyrene in isolated rat hepatocytes. Carcinogenesis 16:2707–15. 10.1093/carcin/16.11.2707 7586190

[10] Prough RA, Patrizi VW, Okita RT, Masters BS, Jakobsson SW (1979) Characteristics of benzo(a)pyrene metabolism by kidney, liver and lung microsomal fractions from rodents and humans. Cancer Res 39:199–206 421229

[11] Thirman MJ, Albrecht JH, Krueger MA, Erickson RR, Cherwitz DL, Park SS, Gelboin HV, Holtzman JL (1994) Induction of cytochrome CYPIA1 and formation of toxic metabolites of benzo[a]pyrene by rat aorta: a possible role in atherogenesis. Proc Natl Acad Sci U S A 91:5397–5401. 10.1073/pnas.91.12.5397 PMC44002 8202497

[12] Lu D, Harvey RG, Blair IA, Penning TM (2011) Quantitation of benzo[a]pyrene metabolic profiles in human bronchoalveolar (H358) cells by stable isotope dilution liquid chromatography-atmospheric pressure chemical ionization mass spectrometry. Chem Res Toxicol 24:1905–1914. 10.1021/tx2002614 PMC3725129 21962213

[13] O’Brien PJ (1991) Molecular mechanisms of quinone cytotoxicity. Chem Biol Interact 80:1–41. 10.1016/0009-2797(91)90029-7 1913977

[14] Bolton JL, Trush MA, Penning TM, Dryhurst G, Monks TJ (2000) Role of quinones in toxicology. Chem Res Toxicol 13:135–160. 10.1021/tx9902082 10725110

[15] Joseph P, Jaiswal AK (1994) NAD(P)H:quinone oxidoreductase1 (DT diaphorase) specifically prevents the formation of benzo[a]pyrene quinone-DNA adducts generated by cytochrome P4501A1 and P450 reductase. Proc Natl Acad Sci U S A 91:8413–8417. 10.1073/pnas.91.18.8413 PMC44616 8078896

[16] Lorentzen RJ, Lesko SA, McDonald K, Ts’o PO (1979) Toxicity of metabolic benzo(a)pyrenediones to cultured cells and the dependence upon molecular oxygen. Cancer Res 39:3194–3198 455303

[17] Flowers-Geary L, Harvey RG, Penning TM (1993) Cytotoxicity of polycyclic aromatic hydrocarbon o-quinones in rat and human hepatoma cells. Chem Res Toxicol 6:252–260. 10.1021/tx00033a002 7686407

[18] Zhu H, Li Y, Trush MA (1995) Characterization of benzo[a]pyrene quinone-induced toxicity to primary cultured bone marrow stromal cells from DBA/2 mice: potential role of mitochondrial dysfunction. Toxicol Appl Pharmacol 130:108–120. 10.1006/taap.1995.1015 7530864

[19] Burdick AD, Davis JW 2nd, Liu KJ, Hudson LG, Shi H, Monske ML, Burchiel SW (2003) Benzo(a)pyrene quinones increase cell proliferation, generate reactive oxygen species, and transactivate the epidermal growth factor receptor in breast epithelial cells. Cancer Res 63:7825–7833 14633709

[20] Shukla H, Chitrakar R, Bibi HA, Gaje G, Koucheki A, Trush MA, Zhu H, Li YR, Jia Z (2020) Reactive oxygen species production by BP-1,6-quinone and its effects on the endothelial dysfunction: Involvement of the mitochondria. Toxicol Lett 322:120–130. 10.1016/j.toxlet.2020.01.011 31953210

[21] Resmish S (1994) Characterization of benzo(a)pyrene quinone-induced toxicity to primary cultured bone marrow stromal cells from DBA/2 mice. PhD. Thesis. The Johns Hopkins University 10.1006/taap.1995.1015 7530864

[22] He H, Wang X, Gorospe M, Holbrook NJ, Trush MA (1999) Phorbol ester-induced mononuclear cell differentiation is blocked by the mitogen-activated protein kinase kinase (MEK) inhibitor PD98059. Cell Growth Differ 10:307–315 10359012

[23] Rickwood D, Wilson MT, Datley-Usmar VM (1987) Isolation and characteristics of intact mitochondria. In: Darley-Usmar VM, Rickwood D, Wilson MT (eds) Mitochondria: A Practical Approach. IRL Press, Oxford, UK, pp 1–16

[24] Bradford MM (1976) A rapid and sensitive method for the quantitation of microgram quantities of protein utilizing the principle of protein-dye binding. Anal Biochem 72:248–254. 10.1016/0003-2697(76)90527-3 942051

[25] Li Y, Zhu H, Trush MA (1999) Detection of mitochondria-derived reactive oxygen species production by the chemilumigenic probes lucigenin and luminol. Biochim Biophys Acta 1428:1–12. 10.1016/s0304-4165(99)00040-9 10366754

[26] Cain K, Skilleter DN (1987) Preparation and use of mitochondria in toxicological research. In: Snell K, Mullock B (eds) Biochemical Toxicology: A Practical Approach. IRL Press, Oxford, UK, pp 217–254

[27] Roubaud V, Sankarapandi S, Kuppusamy P, Tordo P, Zweier JL (1997) Quantitative measurement of superoxide generation using the spin trap 5-(diethoxyphosphoryl)-5-methyl-1-pyrroline-N-oxide. Anal Biochem 247:404–11. 10.1006/abio.1997.2067 9177705

[28] Li Y, Zhu H, Kuppusamy P, Roubaud V, Zweier JL, Trush MA (1998) Validation of lucigenin (bis-N-methylacridinium) as a chemilumigenic probe for detecting superoxide anion radical production by enzymatic and cellular systems. J Biol Chem 273:2015–2023. 10.1074/jbc.273.4.2015 9442038

[29] Li Y, Stansbury KH, Zhu H, Trush MA (1999) Biochemical characterization of lucigenin (Bis-N-methylacridinium) as a chemiluminescent probe for detecting intramitochondrial superoxide anion radical production. Biochem Biophys Res Commun 262:80–87. 10.1006/bbrc.1999.1174 10448072

[30] Davies KJ, Doroshow JH (1986) Redox cycling of anthracyclines by cardiac mitochondria. I. Anthracycline radical formation by NADH dehydrogenase. J Biol Chem 261:3060–3067 3456345

[31] T D (1992) The mitochondrion in Health and Disease. VCH Publishers, New York

[32] Majander A, Finel M, Wikstrom M (1994) Diphenyleneiodonium inhibits reduction of iron-sulfur clusters in the mitochondrial NADH-ubiquinone oxidoreductase (complex I). J Biol Chem 269:21037–21042 8063722

[33] Treberg JR, Quinlan CL, Brand MD (2011) Evidence for two sites of superoxide production by mitochondrial NADH-ubiquinone oxidoreductase (complex I). J Biol Chem 286:27103–27110. 10.1074/jbc.M111.252502 PMC3149303 21659507

[34] von Jagow G, Engel WD (1981) Complete inhibition of electron transfer from ubiquinol to cytochrome b by the combined action of antimycin and myxothiazol. FEBS Lett 136:19–24. 10.1016/0014-5793(81)81206-9 7319059

[35] Turrens JF, Alexandre A, Lehninger AL (1985) Ubisemiquinone is the electron donor for superoxide formation by complex III of heart mitochondria. Arch Biochem Biophys 237:408–414. 10.1016/0003-9861(85)90293-0 2983613

[36] Forman HJ, Boveris A (1982) Superoxide and hydrogen peroxide in mitochondria. In: Pryor WA (ed) Free Radicals in Biology. Academic Press, New York, NY, pp 65–90

